# Beyond the Usual Suspects: Unilateral Optic Disc Edema as a Rare Initial Sign of Vogt-Koyanagi-Harada Syndrome

**DOI:** 10.22336/rjo.2025.44

**Published:** 2025

**Authors:** Vipin Rana, Vikas Sharma, Kanwaljeet Singh, Amit Nandan Tripathi, Ranjit Goenka, Ashish Markan

**Affiliations:** 1 Department of Ophthalmology, Command Hospital (Eastern Command), Kolkata, India; 2 Department of Ophthalmology, Air Force Central Medical Establishment (AFCME), Subroto Park, New Delhi, India; 3 Department of Pathology, Command Hospital, (Eastern Command), Kolkata, India; 4 Department of Ophthalmology, All India Institute of Medical Sciences (AIIMS), New Delhi, India

**Keywords:** Vogt-Koyanagi-Harada syndrome, optic disc edema, optic neuritis, multimodal imaging, VKH = Vogt-Koyanagi-Harada Syndrome, FFA = Fundus fluorescein angiography, ICG = Indocyanine green angiography, EDI-OCT = Enhanced depth imaging-Optical coherence tomography, VEP = Visual evoked potential, RAPD = Relative apparent pupillary defect, MRI = Magnetic resonance imaging, ACE = Angiotensin-converting enzyme, CECT = Contrast-enhanced computed tomography

## Abstract

**Objective:**

To report a case of unilateral optic disc edema as a rare initial presentation of Vogt-Koyanagi-Harada (VKH) syndrome and emphasize the importance of early diagnosis using advanced imaging and cerebrospinal fluid analysis.

**Case Presentation:**

We present the case of a 23-year-old male who initially presented with unilateral optic disc edema, retro-orbital pain, and headache, progressing to bilateral involvement with serous retinal detachments. Advanced imaging, including fundus fluorescein angiography (FFA), Indocyanine green angiography (ICG), and Enhanced depth imaging-Optical coherence tomography (EDI-OCT), revealed hallmark findings of VKH, such as choroidal granulomas and increased choroidal thickness. Cerebrospinal fluid analysis confirmed pleocytosis and melanin-laden macrophages, which helped to establish the diagnosis. The patient was treated with high-dose intravenous corticosteroids and azathioprine, with significant improvement.

**Discussion:**

VKH progresses through prodromal, acute uveitic, chronic, and recurrent phases. Although typically presenting with panuveitis, isolated optic disc edema as an initial sign is rare. Early diagnosis requires multimodal imaging and cerebrospinal fluid analysis to differentiate VKH from other inflammatory and infectious aetiologies.

**Conclusion:**

This case highlights the importance of considering VKH in patients presenting with disc edema, whether unilateral or bilateral, particularly when accompanied by vitreous cells. Advanced ocular imaging and thorough systemic evaluation are critical for early diagnosis. Prompt treatment can prevent progression to chronic disease and irreversible vision loss.

## Introduction

Vogt-Koyanagi-Harada disease (VKH) is an autoimmune syndrome that primarily targets melanocyte-rich tissues, including the central nervous system, eyes, ears, and skin. It is triggered by T-cell-mediated autoimmune dysregulation, often associated with the HLA-DRB1*0405 allele. While VKH typically presents with bilateral panuveitis and systemic involvement, it may occasionally manifest with atypical features, such as isolated optic disc edema [1-2]. VKH should be considered in patients presenting with disc edema, headache, and prodromal symptoms, even in the absence of overt ocular inflammation. Here, we present a case of VKH syndrome that initially presented as unilateral optic disc edema and subsequently progressed to bilateral involvement.

## Case report

**Fig. 1:**
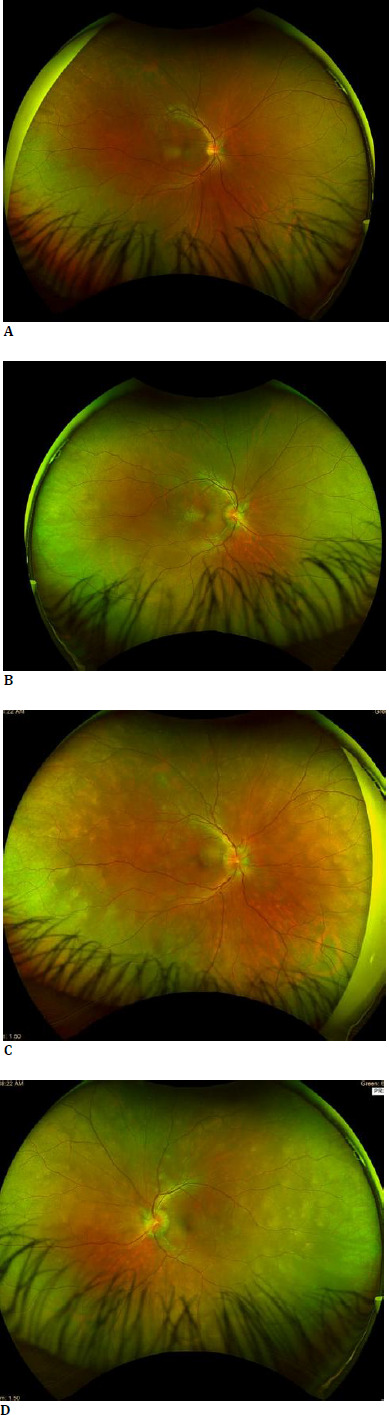
Optos fundus imaging of the patient on presentation. Right eye (**A**) showing normal optic nerve head and peripheral fundus. Left eye (**B**) shows blurred nasal optic disc margin with hyperemia suggestive of optic disc edema. Following a one-month follow-up, the fundus pictures of the right eye (**C**) and left eye (**D**) revealed bilateral optic disc edema and peripheral fundus granularity

A 23-year-old male presented with retro-orbital pain in the left eye and a recent history of headache lasting 2-3 days. Visual acuity was 20/20 bilaterally, and the anterior segment examination was unremarkable. Posterior segment examination revealed isolated left optic disc edema (**Fig. 1A, B**), while the remainder of the ocular examination was normal. His colour vision, contrast sensitivity, and pupillary responses were intact. Initial investigations, including fundus fluorescein angiography (FFA), visual evoked potential (VEP), and visual field testing, were normal. Magnetic resonance imaging (MRI) of the brain and optic nerves revealed swelling in the intrabulbar portions of both optic nerves, with no evidence of increased intracranial pressure or intracranial space-occupying lesions. Given the diagnostic uncertainty, we considered pseudo disc edema and opted to monitor the patient without initiating treatment. Our rationale was that if this were pseudo-disc edema, the condition would likely remain stable, whereas an evolving disease process would manifest with additional features over time.

One month later, the patient returned with complaints of bilateral visual blurring. Visual acuity had reduced to 20/30 in the right eye and 20/40 in the left. The anterior segment remained unremarkable, but trace vitreous cells were noted in the right eye and grade 1+ cells in the left. Fundus examination revealed bilateral optic disc edema and peripheral fundus granularity (**Fig. 1C, D**). Based on these new findings, our differential diagnosis expanded to include sarcoidosis, Vogt-VKH syndrome, syphilis, tuberculosis, bilateral optic neuritis, and other causes of optic disc edema.

A comprehensive workup was conducted, including complete blood count, liver and renal function tests, serum electrolytes, Mantoux test, serum angiotensin-converting enzyme (ACE) levels, vitamin B1, B2, B12 levels, antinuclear antibodies, fundus autofluorescence, erythrocyte sedimentation rate, C-reactive protein, and infectious disease screenings (TPHA, rapid syphilis antibody, HBsAg, HCV, HIV). Imaging studies included contrast-enhanced computed tomography (CECT) of the chest, a repeat MRI of the brain, and imaging of the optic nerves. All results were normal except for swelling in the intrabulbar portions of both optic nerves, with no evidence of raised intracranial pressure or intracranial lesions.

**Fig. 2:**
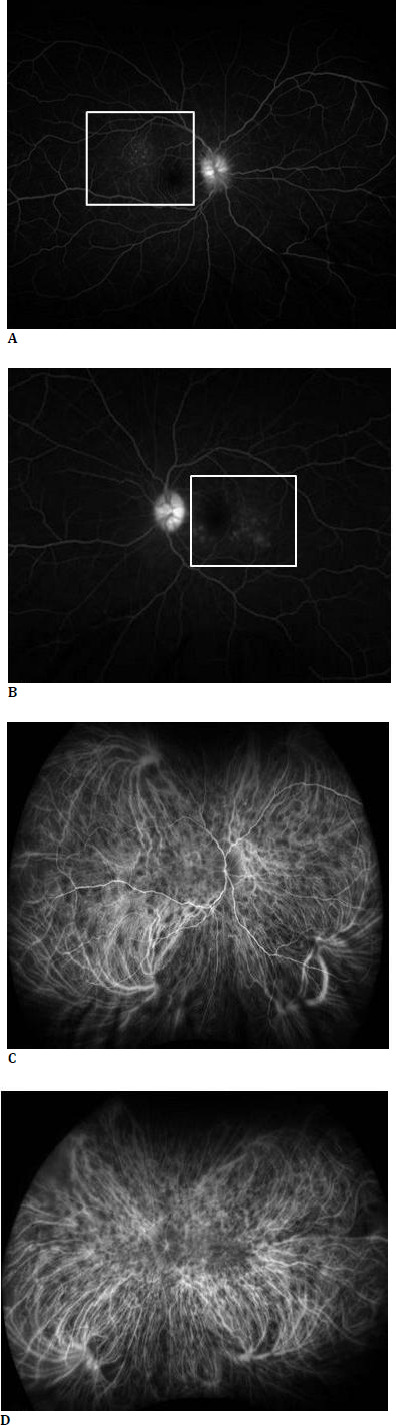
Fundus fluorescein angiogram (FFA) of right eye (**A**) and left eye (**B**) revealed bilateral serous retinal detachments with pinpoint hyperfluorescence, pooling, and a “starry sky” appearance in the late phase. Indocyanine green angiography (ICG) of the right eye (**C**) and left eye (**D**) showed multiple hypocyanescent areas consistent with choroidal granulomas

**Fig. 3:**
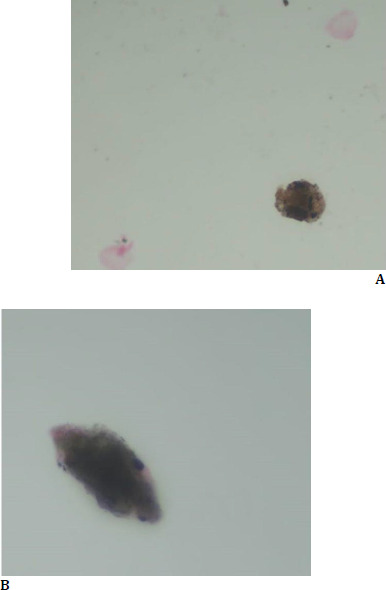
Cerebrospinal fluid sampling showing melanin-laden macrophages (**A**) and an enlarged view (**B**)

Ocular imaging with FFA revealed bilateral serous retinal detachments, characterized by pinpoint hyperfluorescence in the early phase, pooling, and a “starry sky” appearance in the late phase (**Fig. 2A, B**). Indocyanine green angiography (ICG) showed multiple hypocyanescent areas consistent with choroidal granulomas (**Fig. 2C, D**). Enhanced depth imaging-optical coherence tomography (EDI-OCT) revealed increased choroidal thickness and serous detachment bilaterally. Cerebrospinal fluid analysis revealed pleocytosis with melanin-laden macrophages (**Fig. 3A, B**), confirming a VKH diagnosis in the acute uveitic stage. Systemic evaluation for the other organ involvement was negative.

The patient was started on intravenous methylprednisolone (1 mg/kg) and transitioned to steroid-sparing immunosuppressive therapy with azathioprine, in consultation with rheumatology. His symptoms improved, and he was discharged in stable condition.

## Discussion

This case highlights the importance of considering VKH syndrome as a differential diagnosis in patients presenting with disc edema, particularly when accompanied by headache and prodromal symptoms. VKH is a rare, autoimmune inflammatory disorder that primarily affects melanocyte-rich tissues such as the eyes, skin, auditory system, and central nervous system [3,4]. Although VKH is uncommon and typically presents with panuveitis, it can, in rare cases, initially manifest with isolated optic nerve involvement. This makes it a challenging diagnosis, particularly when it presents in an atypical manner. Failure to recognize VKH in such cases may delay diagnosis and treatment, potentially leading to irreversible vision complications.

The patient’s initial presentation of unilateral optic disc edema with retrobulbar pain raised suspicion for optic neuritis. However, the absence of a relative afferent pupillary defect (RAPD), normal colour vision and contrast sensitivity, and normal VEP findings argued against this diagnosis. As the disease progressed with bilateral involvement and serous retinal detachments, inflammatory aetiologies such as VKH, sarcoidosis, syphilis, tuberculosis, and autoimmune optic neuropathy became more likely.

VKH disease typically has four clinical phases: Prodromal Phase: This initial phase can resemble a flu-like viral prodrome, characterized by symptoms such as headache, fever, malaise, meningismus (neck stiffness), and, occasionally, mild hearing disturbances (tinnitus or dysacusis). Neurological symptoms, such as meningitis, may also appear at this stage. Acute Uveitic Phase: Following the prodromal phase, there’s a sudden onset of bilateral, granulomatous panuveitis. Patient typically presents with ocular symptoms, viz blurred vision, photophobia, pain, and floaters. Chronic (Convalescent) Phase: Melanocyte-containing organs get involved in this stage. Vitiligo, poliosis (whitening of eyelashes), and alopecia (hair loss) occur due to the destruction of melanocytes in these areas. Recurrent (Chronic Recurrent) Phase: In this phase, uveitis can recur, usually with less intensity, but it can still lead to complications such as cataracts, glaucoma, and choroidal neovascularization [5].

Advanced imaging modalities played a pivotal role in distinguishing VKH from other causes of optic disc edema. EDI-OCT confirmed serous retinal detachments and choroidal thickening, FFA demonstrated multiple pinpoint leakages, and ICG identified choroidal granulomas, findings highly consistent with VKH. Furthermore, CECT and MRI excluded neurological causes such as cerebral venous thrombosis, optic disc drusen, intracranial space-occupying lesions, and infiltrative malignancies [6-8].

Lumbar puncture findings of cerebrospinal fluid pleocytosis and melanin-laden macrophages, combined with the imaging results, confirmed VKH in its acute phase. The prodromal symptoms of headache and fever further aligned with the inflammatory phase of VKH, underscoring the importance of a thorough symptom history in patients with disc edema.

Early intervention with corticosteroids and immunosuppressive therapy is crucial to prevent progression to chronic disease and serious ocular complications. Studies show that initiating combination therapy within 2-3 weeks of symptom onset significantly improves outcomes and reduces the likelihood of chronicity [9]. In this case, high-dose intravenous methylprednisolone and azathioprine were effective, demonstrating the importance of aggressive immunosuppressive therapy in managing VKH.

## Conclusion

This case highlights the importance of considering VKH syndrome in patients presenting with optic disc edema, whether unilateral or bilateral, especially when accompanied by vitreous cells. Advanced ocular imaging and thorough systemic evaluation are critical for early diagnosis. Timely treatment with corticosteroids and immunosuppressive therapy can prevent progression to chronic disease and irreversible vision loss.
